# Symplastic guard cell connections buffer pressure fluctuations to promote stomatal function in grasses

**DOI:** 10.1111/nph.70009

**Published:** 2025-02-15

**Authors:** Matthew J. Wilson, Shauni McGregor, Clinton H. Durney, Melissa Tomkins, Jodie Armand, Richard S. Smith, Julie E. Gray, Richard J. Morris, Andrew J. Fleming

**Affiliations:** ^1^ Plants, Photosynthesis and Soils, School of Biosciences University of Sheffield, Western Bank Sheffield S10 2TN UK; ^2^ Computational and Systems Biology John Innes Centre Norwich Research Park Norwich NR4 7UH UK

**Keywords:** biomechanics, computational modelling, grass, guard cell, imaging, stomata, symplastic connections, turgor

## Abstract

- Stomata regulate plant gas exchange via repeated turgor‐driven changes of guard cell shape, thereby adjusting pore apertures. Grasses, which are among the most widespread plant families on the planet, are distinguished by their unique stomatal structure, which is proposed to have significantly contributed to their evolutionary and agricultural success. One component of their structure, which has received little attention, is the presence of a discontinuous adjoining cell wall of the guard cell pair.
- Here, we demonstrate the presence of these symplastic connections in a range of grasses and use finite element method simulations to assess hypotheses for their functional significance.
- Our results show that opening of the stomatal pore is maximal when the turgor pressure in dumbbell‐shaped grass guard cells is equal, especially under the low pressure conditions that occur during the early phase of stomatal opening. By contrast, we demonstrate that turgor pressure differences have less effect on the opening of kidney‐shaped guard cells, characteristic of the majority of land plants, where guard cell connections are rarely or not observed.
- Our data describe a functional mechanism based on cellular mechanics, which plausibly facilitated a major transition in plant evolution and crop development.

## Introduction

Stomata are epidermal pores that form the cellular interface between plants and their environment. Each pore is formed by a pair of guard cells (GCs), which act to dynamically regulate pore aperture, and thus the flux of gases across the epidermis, via repeated turgor‐driven movements. Stomatal adjustments are essential for regulating transpiration and balancing the uptake of CO_2_ for photosynthesis with the loss of water (Hetherington & Woodward, 2003; Lawson & Matthews, 2020). The majority of plant families have stereotypically kidney‐shaped GCs, whereas the grasses (also known as the Poaceae/Gramineae) that include major crop species exhibit a novel stomatal morphology with dumbbell‐shaped guard cells that are flanked by specialised lateral subsidiary cells (SCs) (Stebbins & Shah, 1960; Nunes *et al*., 2020). The SCs are proposed to improve the speed of stomatal opening and closing by providing a local pool of ions and metabolites required to drive changes in GC turgor pressure (Raschke & Fellows, 1971; Büchsenschütz *et al*., 2005; Schäfer *et al*., 2018; Gray *et al*., 2020). The unique structure of grass stomata is believed to elevate their sensitivity to environmental fluctuations and to enhance the water use efficiency of grasses (Franks & Farquhar, 2007; McAusland *et al*., 2016).

Grass GCs have distinct domains: A central rod region connects a pair of bulbous ends at the proximal and distal poles of the cell to form an approximate dumbbell shape (Aylor *et al*., 1973; Jaafar & Anderson, 2024). Cell wall thickness differs significantly between these domains, with the wall in the central rod region or canal being much thicker than that of the bulbous ends (Stebbins & Shah, 1960; Durney *et al*., 2023; Gkolemis *et al*., 2023). The cell walls of grass GCs are further specialised, having specific composition and cell wall anisotropy patterns (Rui *et al*., 2018). Recent studies support the suggestion that this unique geometry of grass GCs, specialised cell walls, and the presence of a reciprocal pressure exchange system with the subsidiary cells are important for the function of grass stomata and thus improved performance over a two‐celled kidney‐shaped system (Raissig *et al*., 2017; Durney *et al*., 2023; Gkolemis *et al*., 2023; Liu *et al*., 2024).

Our recent studies in this area have used computational modelling to simulate the effect of turgor change on the function of grass (dumbbell‐shaped) and eudicot (kidney‐shaped) GCs. Whilst GCs are often assumed to operate as symplastically isolated units, each separately generating and maintaining turgor to drive the cell shape changes required for stomatal pore opening and closure, differences in turgor pressure between GCs have not been explored in these models (Carter *et al*., 2017; Durney *et al*., 2023). Indeed, there is substantial evidence in the literature to support the lack of linkages between adjacent GCs and SCs (Wille & Lucas, 1984). For example, microinjection experiments in onion and *Commelina communis* show that dye does not move out of a mature GC, whilst easily passing between adjacent epidermal pavement cells and between guard mother cells and immature GCs where a pore is yet to form (Palevitz & Hepler, 1985). In grasses, similar experiments show that dumbbell‐shaped GCs are not linked to their adjacent subsidiary cells (Erwee *et al*., 1985; Mumm *et al*., 2011), and no plasmodesmata have been observed in ultrastructural analyses between GCs and SCs (Brown & Johnson, 1962; Srivastava & Singh, 1972).

With respect to the GC pairs themselves, the presence of symplastic connections has been observed in some (but not all) species of earlier diverging branches of the plant evolutionary tree, with discontinuous cell walls identified in one horsetail (Cullen & Rudall, 2016), and GCs in two fern species have been shown to be symplastically connected (Voss *et al*., 2018). An extreme and unusual example of a conjoined GC pair has been identified in the moss *Funaria hygrometrica*, which has a large gap in the ventral wall between the GCs as a result of incomplete cytokinesis (Merced & Renzaglia, 2014). Interestingly, ultrastructural analyses of GCs in the grass relative *Flagellaria indica* did not identify the presence of a discontinuous cell wall (Sack, 1994). In eudicots and grasses, although there are several reports of the presence of gaps in cell walls between adjoining GCs dating from the 1960s to 1970s (Brown & Johnson, 1962; Pickett‐Heaps, 1967; Kaufman *et al*., 1970; Srivastava & Singh, 1972; Galatis, 1980), as well as dye‐loading experiments showing that mature maize GCs are symplastically connected (Mumm *et al*., 2011), these observations seem to have been mostly forgotten in the more recent literature (although have of late been mentioned; Spiegelhalder & Raissig, 2021). This has led to a widely accepted view that the most commonly observed situation of complete GC symplastic isolation seen in eudicot kidney‐shaped GCs (Pallas & Mollenhauer, 1972; Willmer & Sexton, 1979; Wille & Lucas, 1984; Zhao & Sack, 1999) is a general feature of stomata.

We report here on a series of experiments which, first, verify the occurrence of large symplastic connections between adjacent GCs in all grass species tested. Second, for the first time, we use computational simulations to explore the potential outcome of such symplastic connections on stomatal biomechanics by independently varying individual guard cell pressures, creating local asymmetries in GC pressure. In particular, we test the hypothesis that GC pairs acting as a single osmotic unit provide functional advantages to grass stomata. Finally, we investigate potential reasons why GC pairs in eudicot (kidney‐shaped) stomata do not generally show such symplastic connections. Our data reveal that a long‐observed but frequently overlooked element of grass GC structure may play an important role in enhancing stomatal function.

## Materials and Methods

### Plant material and growth conditions

*Brachypodium* *distachyon* (L.) *P.Beauv*. (Bd21‐3), barley (*Hordeum vulgare* (L.), cv Golden Promise), maize (*Zea mays* (L.), cv Delprim), and onion (*Allium cep* (L.), cv White Lisbon) seed were germinated in Levington M3 compost in a controlled environment growth chamber (16 h : 8 h, 21°C : 16°C, light : dark, 400 μmol m^−2^ s^−1^ PPFD, 60% relative humidity). After 7–9 d, seedlings were transplanted to larger pots containing a 3 : 1 (v/v) ratio of M3 and perlite, plus 5 g of solid slow‐release fertiliser and kept well‐watered. Leaf tissue was harvested from the fully expanded 5^th^ leaf.

### Transmission electron microscopy

Leaf tissue was cut into strips, fixed in 2% glutaraldehyde solution (v/v) in 0.1 M sodium cacodylate buffer under vacuum, and washed twice with PBS before secondary fixation with 2% aqueous osmium tetroxide solution (v/v) for 2 h at ambient temperature. After three washes with PBS, samples were dehydrated via an ethanol series (30%, 50%, 70%, 90%, 100%, 100%, 30 min per change) and left in 100% ethanol overnight. Samples were transferred to propylene oxide for two changes, each for 15 min, and infiltrated with Araldite CYC212 resin (Agar Scientific, Rotherham, UK; 50% mixture of resin in propylene oxide, overnight; 100% resin, overnight), embedded in fresh resin, and left to harden for 72 h at 60°C.

Ultrathin (70–90 nm) sections were cut with a Reichert‐Jung Ultracut E ultramicrotome fitted with a DiATOME diamond knife, transferred to nickel grids, stained with a 3% aqueous uranyl acetate solution for 30 min, destained with water for 30 min, and finally stained with Reynold's lead citrate for 5 min. Sections were examined using an FEI Tecnai T12 Spirit Transmission Electron Microscope at an accelerating voltage of 80 kV. Electron micrographs were taken using a Gatan digital camera.

### Cellulose microfibril orientation

GC cell wall anisotropy was examined in barley epidermal peels and onion leaf sections. The abaxial epidermis of the first fully expanded leaf (7–9 DAS) of barley was isolated from the subtending mesophyll by peeling, and onion leaf tissue was harvested from the fifth fully expanded leaf. The isolated epidermis or leaf tissue was immediately floated on a resting buffer (50 mM KCl, 10 mM MES, 5 mM KNO3, pH 6.2) and incubated at 21°C for 2 h, with the addition of the bifluorescent cellulose‐specific stain 0.1% Pontamine Fast Scarlet (PFS) for the last 30 min. After rinsing in 1× PBS, samples were mounted using resting buffer in round glass‐bottomed dishes and imaged using a Zeiss LSM800 AiryScan confocal microscope at maximum scan speed, and pinhole size 1 AU. Barley samples were oriented so that leaf vasculature (and thus stomatal long axis) was either aligned parallel or perpendicular to the polarization angle of the excitation beam. Onion samples were oriented so that the long axis of the GCs was aligned either *c*. +45° or −45° from the polarization angle of the laser. 3D *z*‐stacks of individual stomates were collected, with a step size of 0.3 μm. After this first stack had been acquired, the sample was rotated by 90° on the stage and the process repeated. To reduce any impacts of bleaching on the interpretation of fluorescence intensity data, the order in which stacks were taken was randomised. Sixteen barley stomata and 10 onion stomata were imaged across five and three biological replicates, respectively. Images were processed via AiryScan processing before being converted into .tiff files. Images of each of the orientations were initially approximately manually aligned before accurate computational 3D registration using the ImageJ plugin Fijiyama (Fernandez & Moisy, 2021). Maximum intensity projections were produced in Fiji (Schindelin *et al*., 2015) using aligned images. Signal intensity differences resulting from the bifluorescent nature of PFS enabled gross patterns of cellulose microfibril orientation in guard cells and subsidiary cells to be observed.

### Confocal imaging and segmentation

Barley leaf tissue sections (1 cm^2^) were excised from the middle third of the fully expanded fifth leaf and incubated for 2 h at 21°C in 50 mM KCl, 10 mM MES, 5 mM KNO3, pH 6.2 buffer plus either 10 μM fusicoccin (Fc) or 10 μM abscisic acid (ABA) to stimulate stomatal opening and closure. After treatment, samples were immediately submerged in a fixative of 3 : 1 ethanol : acetic anhydride (v/v) before vacuum infiltration for 1 h. Samples were left in the fixative at 4°C for 48 h, rinsed in 50% ethanol, and transferred to 70% ethanol for storage. Onion leaf sections were cut from fully expanded fifth leaf and immediately submerged in 3 : 1 ethanol : acetic anhydride (v/v), and fixed and stored in the same way.

Barley and onion samples were prepared for confocal laser scanning microscopy as previously described (Durney *et al*., 2023). Leaf samples were removed from 70% ethanol, briefly treated with chloroform to remove epicuticular waxes, progressively rehydrated via an ethanol series before bleaching and starch digestion via amylase treatment. Cell walls were stained with pseudo‐Schiff propidium iodide (PI) and samples cleared in chloral hydrate + glycerol, mounted on slides in Hoyer's solution, kept in the dark, and imaged within 3 d of mounting to avoid photobleaching or desiccation.

Images of individual barley stomata were collected using an Olympus FLUOVIEW FV1000 confocal system with a 40× oil immersion objective (UPlanApo 40×, NA: 1.0). PI was excited using a 561 nm HeNe561 diode laser. Scan resolution was 640 × 640 pixels with a pixel dwell time of 12 μs/pixel. No averaging was performed, and bidirectional scanning was enabled. *Z*‐step was set at 0.3 μm to aid segmentation accuracy. Each stack began a few microns above the stomatal complex and ended a few microns below, in the substomatal cavity. Twenty‐four open and 24 closed stomata were imaged across six biological replicates for each treatment.

Onion images were collected using a Zeiss LSM800 AiryScan confocal microscope in LSM mode using a 20× air objective (Plan‐Apochromat 20×, NA: 0.8). Cell walls labelled with PI were visualised using the 561 laser and a 488/561 dichroic mirror. Resolution was set at 488 × 488 pixels and scan speed was set to maximum. Pinhole size was maintained at 1 AU. Step size was 0.3 μm, and stacks were initiated above, and completed below, each stomate. Images were subjected to AiryScan Processing (Zen; Zeiss). Eighteen stomata were imaged across three biological replicates.

To confirm the presence (or absence) and to measure the maximum size of the connections between GCs as indicated from Transmission Electron Microscopy (TEM) analysis, confocal images were resliced to observe the stack in the XZ plane. In stomata in which a discontinuous shared cell wall between GCs could be observed, the slice containing the largest gap in the cell wall was identified and the maximum width measured. Reslicing of confocal images and length measurements were carried out in Fiji (Schindelin *et al*., 2015). Analysis was carried out on the confocal images directly underpinning both models (24 closed barley stacks, six biological reps; 18 closed onion stacks, three biological reps).

Segmentation was performed using MorphoGraphX (MGX) (Strauss *et al*., 2022). Images were converted into .tiff files using Fiji before being imported into MGX and brightened using the ‘Stack/Filter/Brighten Darken’ process. A difference‐of‐Gaussians filter was applied to image stacks of barley stomata using the process ‘Stack/Filter/Diff Gaussians’ to emphasise edges of the highly fluorescent signal in the rod region, before the utilisation of the built‐in, pretrained CNN for cell boundary prediction using the process ‘Stack/CNN/UNet3D’ (Vijayan *et al.*, 2021). For onion images, the CNN was used directly without the difference‐of‐Gaussians filter as cell boundaries were detectable without this. Stacks were further blurred twice using the process ‘Stack/Filter/Gaussian Blur Stack’ with a radius of 0.3 μm, and 3D images segmented using the ‘Stack/ITK/Segmentation/ITK Watershed Auto Seeded’ function with a threshold of 500. Since the watershed algorithm automatically identifies the midpoint of the cell wall signal, the segmentation line is shifted slightly inwards from the true boundary. Labels other than those corresponding to desired cells (i.e. epidermal pavement cells, mesophyll cells, and intercellular air spaces) were manually removed from each stack, whilst any oversegmentation errors in the guard cells and subsidiary cells were corrected where required. Mesh files of cell geometry were generated using ‘Mesh/Creation/Marching Cubes 3D’ with a cube size of 1 μm and three smooth passes. Where required to better capture cell geometry, meshes were smoothed further using the process ‘Mesh/Structure/Smooth Mesh’. Onion meshes were rescaled in the *Z*‐direction as described previously (Diel *et al*., 2020) to account for refractive index differences associated with image acquisition. Mesh files were used directly to inform finite element analysis in MorphoMechanX. Cell surface area and volume, and geometry differences between GCs were extracted from meshes using the process ‘Mesh/HeatMap/Heatmap Classic’. Supplementary videos showing the XZ plane along the length of the stomatal complex were also created using MGX.

### Computational modelling

Mechanical models were formulated within the MorphoMechanX framework (https://www.morphomechanx.org/) utilising previously established methods (Hofhuis *et al*., 2016; Mosca *et al*., 2017; Durney *et al*., 2023). These models are predicated on parameters for geometry and material properties to simulate the elastic behaviour of the cell wall under an internal load. The Finite Element Method (FEM) was employed to determine mechanical equilibrium under the application of turgor pressure to the stomatal complex walls.

The initial geometries for the FEM simulations were obtained by extracting surface meshes from 3D confocal images using MorphoGraphX of closed stomata as described in the previous section. For barley, a total of 24 meshes from six biological replicates were used, whilst 18 meshes from three biological replicates were used for onion. A triangular surface mesh representing the cell and cell wall geometries was generated from the segmented image. Each cell is formed by closed surfaces composed of triangles, with shared triangles and vertices on the walls between cells. These triangle positions were used to create triangular membrane elements, which were assigned specific thicknesses and material properties. TEM analysis of barley GCs indicated that the cell wall in the rod region is approximately three times thicker than in the bulbous ends (Durney *et al*., 2023). The GC well of the onion cells was set to uniform thickness throughout (Supporting Information Table S1; Fig. S2c,g).

To provide realistic geometries for GCs, meshes were derived from fixed samples of either barley or onion. Given that the stomatal complexes of grasses are arranged in stratified files and showed no width variation during opening and closing, the lateral outer boundary of the subsidiary cells was fixed in space (Durney *et al*., 2023). The outer boundary of the onion cells was fixed at the poles, consistent with Carter *et al*. (2017). A schematic implementation of boundary conditions for each species is given in Fig. S2(d,h). The cell walls were modelled as a transverse isotropic Saint–Venant Kirchoff material with two Young's moduli (E1/E3, E2). The Poisson ratios for different directions were reduced to a single parameter to approximate the compressibility as if the material were isotropic. Young's modulus, E2, defines the material stiffness in the direction of the primary orientation of cellulose microfibrils, which are longitudinally oriented in barley stomata and circumferentially oriented in onion stomata, as depicted in Fig. S2(b,f). Young's modulus, E1/E3, defines stiffness in the orthogonal direction. Mechanical properties for the GCs and subsidiary cells are the same as reported in Durney *et al*. (2023). Mechanical parameters for onion were chosen such that the meshes maintained their geometry without experiencing any significant deformation, thereby matching real‐world behaviour (Fig. S3). All mechanical parameters are summarised in Table S1. In the barley model, SC pressure was fixed to remove this as a variable and simplify the model, whilst also enabling comparison between dumbbell‐ and kidney‐shaped models. GC turgor pressures were deliberately selected to span and extend beyond typical physiological ranges for GC pressures, previously measured at 0–5 MPa for a range of species including wheat (Franks & Farquhar, 2007). This choice was made to explore a broader parameter space and to explore what would happen in the most extreme scenarios where GC pressures could not equalise. The sum of the GC pressures was fixed at each value from 2 to 9 MPa in increments of 1 MPa, and the individual GC pressures were determined by varying the proportion of one GC's pressure from 10 to 90% of the total pressure in increments of 10%. A scenario with the pressure split 50 : 50 across the GC pair was equivalent to simulating the presence of gaps, whereas each other pressure ratio simulated the GC pair without gaps. Turgor pressure was applied as a load normal to the triangular elements, and mechanical equilibrium was found using a pseudo time‐stepping method, as described in Mosca *et al*. (2017).

The model's output includes the deformation, stress, and strain profiles of the stomatal complex, which are influenced by the unique stomatal geometry, mechanical parameters, and turgor pressures chosen. This allows for geometrical features such as pore area, width, and length, as well as stomatal complex length and width to be calculated. Stresses and strains are calculated at the element level, with visualisation using the trace of the respective tensors. We calculated the trace of the Cauchy stress tensor by summing the eigenvalues of the stress tensor for each element. Taking the mean of the top 10% of these values allowed comparison between different pressure ratios across different GC meshes for both species.

### Tissue treatment for onion model validation

Epidermal peels from the fifth fully expanded onion leaf were incubated for 2 h in resting buffer at 21°C under one of the following treatments: under light (400 μmol m^−2^ s^−1^) and supplied with air scrubbed of CO_2_ using soda lime to induce stomatal opening, or dark adapted under ambient CO_2_ to close stomata. After treatment, samples were mounted on slides in buffer before brightfield imaging (Olympus BX51 with DP71 camera). Pore area was measured using Fiji (Schindelin *et al*., 2015). Experimental data (*n* = 28, three biological replicates) were compared with final pore areas simulated from the onion FEM model for each of the individual onion meshes (*n* = 18, three biological replicates).

### Data analysis

Statistical analyses were assessed using a paired (for comparisons of GC geometry within a GC pair) or unpaired (for independent measurements) two‐tailed *t*‐test, or via one‐way ANOVA followed by *post hoc* Tukey's HSD (for comparisons between pressure scenarios) using the statistical package GraphPad Prism. For comparisons within a GC pair, when data were not normally distributed, the alternative Wilcoxon matched‐pairs signed‐rank test was employed. Significance was assumed if *P* ≤ 0.05. Lettering indicates when samples can be distinguished from one another. All error bars represent ± SEM.

## Results

### The bulbous ends of adjacent grass GCs are directly connected to each other via large breaks in the ventral cell wall

**Fig. 1:**
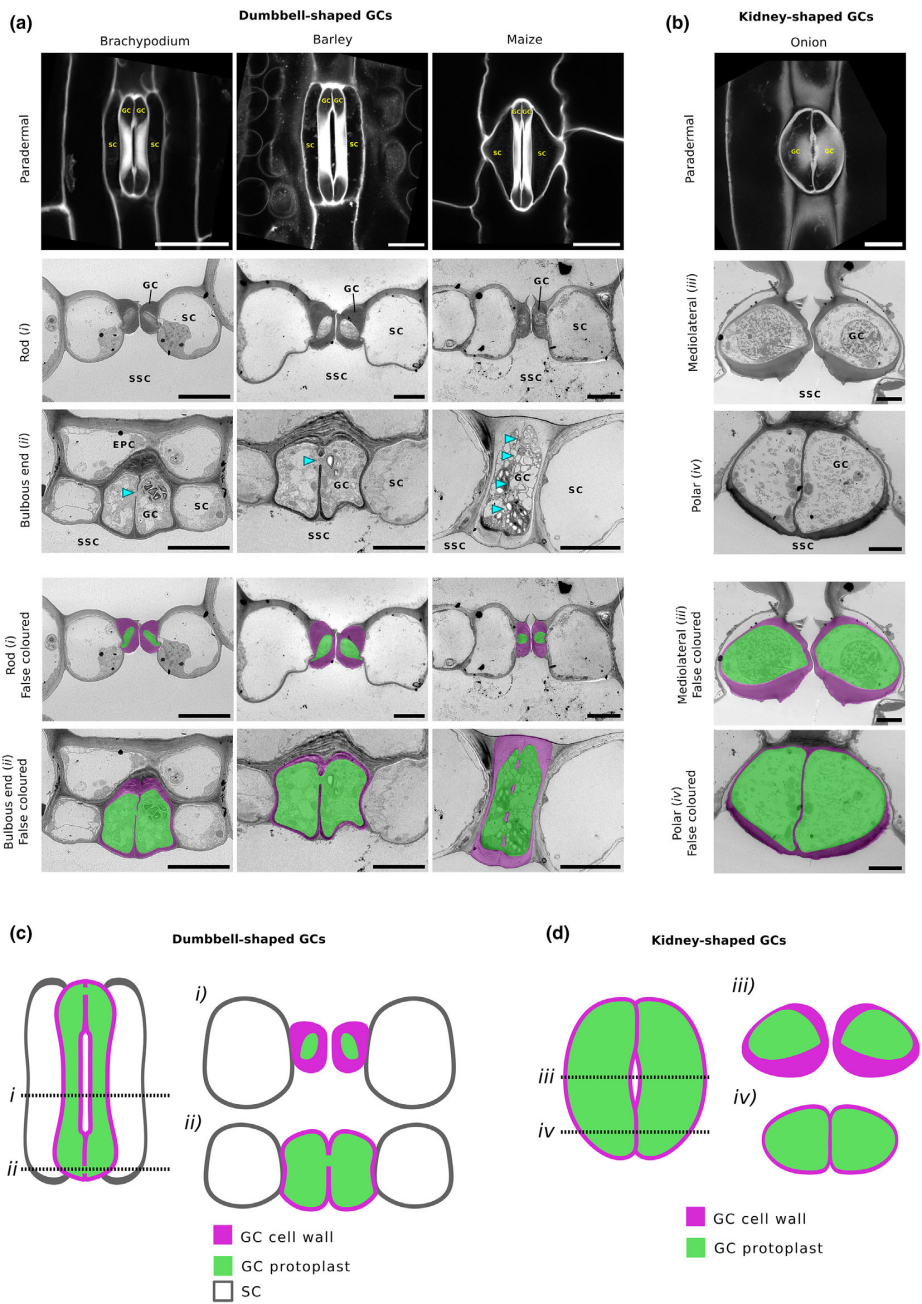
Transmission electron microscopy (TEM) reveals large connections between the bulbous ends of dumbbell‐shaped guard cells (GCs). (a) Fluorescence and TEMs of sections of stomata of the grass species *Brachypodium distachyon*, Barley (*Hordeum vulgare*), Maize (*Zea mays*). Bars, 20 μm. Whilst each species has dumbbell‐shaped GCs flanked by a pair of lateral subsidiary cells (SC), the specific geometry of these differs between grass species, as can be seen when observing stomata in paradermal section. Planes of section for TEM images refer to those as described in (c). Transverse sections taken across the rod region (*i*) show the characteristic thick GC wall associated with grass stomata is present in each of the species. In comparison, the cell wall in the bulbous ends (*ii*) is relatively thinner. In the bulbous ends of each of the grass species, gaps in the cell wall connecting the protoplast of the adjoining GCs in the pair are present (highlighted using cyan arrowheads). Cell wall thickness in SCs is more uniform along the length of the stomatal complex, with no clear differentiation between the rod and bulbous end regions. SSC indicates the substomatal cavity, whilst epidermal pavement cells are labelled EPC. Below, images have been false‐coloured to highlight the GC wall (magenta) and protoplast (green). (b) Paradermal fluorescence and cross‐sectional TEMs of stomata in onion (nongrass monocot). In contrast to grass species, onion stomata are constituted of a pair of kidney‐shaped GCs and lack specialised SCs. Planes of section for TEM images as described in (d). In the mediolateral (*iii*) regions, GCs show variable wall thickness, having thicker ventral, anticlinal, and periclinal cell walls relative to the dorsal wall. In polar regions (*iv*), the GC wall is more uniform in thickness. The ventral wall in this region has no gaps and the GCs within a pair are isolated from one another. Below, images have been false‐coloured to highlight the GC wall (magenta) and protoplast (green). SSC indicates the substomatal cavity. Bars, 5 μm. (c) Grass stomata are composed of a pair of dumbbell‐shaped GCs (green), flanked by a pair of lateral SCs (white). Each GC can be split into distinct regions: a central rod region (transverse section shown by dashed line *i*) adjacent to the stomatal pore – where the cell wall (magenta) is thick – and two polar bulbous ends (transverse section shown by dashed line *ii*), which have a relatively thinner cell wall. In addition to a thinner cell wall, these regions additionally feature gaps in the ventral cell walls of the GCs, symplastically connecting the GC pair. GCs are symplastically isolated from their neighbouring SCs. (d) GCs (green) of onion and many other nongrass species are kidney‐shaped and encircle a central pore. Across the mediolateral axis of the complex (transverse section shown by dashed line *iii*), variable thickness of the cell wall is exhibited. The dorsal wall of the GC can be observed to be thinner than the ventral, periclinal, and anticlinal cell walls. Towards the polar regions of the GCs (transverse section shown by dashed line *iv*), cell wall thickness is more uniform, and the adjacent GCs are symplastically isolated from one another, having complete ventral cell walls in is a general feature of most nongrass species.

To investigate whether grass GCs have gaps in shared ventral walls, and thus confluent protoplasts at their bulbous ends, we used TEM to image cross sections of stomatal complexes of three grass species (*Brachypodium distachyon*, *Hordeum vulgare* (barley), and *Zea mays* (maize)). Stomata in these species have stereotypical dumbbell‐shaped GCs flanked by a pair of lateral subsidiary cells (Fig. 1a). As shown in Fig. 1(a), the central rod regions (transect *i* in Fig. 1c) of the GCs in all species were characterised by the presence of a thick cell wall (highlighted in magenta in the lower panels) encompassing a relatively narrow strip of cytoplasm (highlighted in green in the lower panels). Examination of the bulbous ends found towards the poles of the GCs (transect *ii* in Fig. 1c) highlighted not only their relatively thinner cell walls but also the presence of gaps in the ventral wall between the sister GCs, through which the protoplasts of the adjacent cells are clearly connected (Fig. 1a – cyan arrowheads). This was observed in all three species, though the extent and number of wall perforations differed depending on species and/or GC size. Cytoplasmic connections are highlighted in false‐coloured figure parts with cytosol in green, and cell wall in magenta. These cell wall gaps are in the order of 0.5–1.5 μm in size and are orders of magnitude larger than plasmodesmata.

For comparison with the grass stomata, we analysed stomatal complexes of a nongraminaceous monocot – onion, *Allium cepa*. Onion GCs are kidney‐shaped (Fig. 1a,d) but, in contrast to species such as *Arabidopsis thaliana*, stomata are arranged in cellular files, reminiscent of the epidermal patterning observed in grass species. Our analysis showed that, whilst not as extreme as the variable wall thickness exhibited by grass GCs, in the mediolateral region (transect *iii* in Fig. 1d), the lower ventral wall of the onion GC is thicker than the upper ventral and dorsal walls (Fig. 1b). Towards the polar regions of the complex (transect *iv* in Fig. 1d), the cell wall is more uniform in thickness, and the adjacent GCs are symplastically isolated from one another and their neighbouring cells. The shared ventral cell of the GCs appeared intact, with no visible gaps or plasmodesmata (Fig. 1b). False colouring of the cytosol (green) and cell wall (magenta) locations in Fig. 1(b) illustrates the lack of any observable symplastic connections in onion stomata, in contrast to the situation in grass stomata.

Confocal imaging allowed for the quantification of the frequency and size of cell wall gaps in barley. Of the 24 images of stomata from which 3D guard cell geometry was extracted, a discontinuous cell wall (cell wall stained with propidium iodide) was observed in the bulbous ends of each stomatal complex (Fig. S1a; Video S1). The mean maximum GC gap diameter (Fig. S1c) was measured from XZ projections of the confocal stack. In barley, the mean maximum gap diameter per stomate was found to be 1.45 μm (range = 0.51–3.79 μm). Unlike in the barley stacks, analysis of XY projections showed that large connections between GCs were not observable in any of the 18 confocal images used for mesh generation (Fig. S1b; Video S2). The difference in the presence/absence of gaps between adjacent GCs is summarised in the cartoons shown in Fig. 1(c,d).

Since our aim was to generate mechanistic computational models for stomata representing the two GC types, we also collected data on wall anisotropy (using a fluorescent dye), since cellulose microfibril orientation is often a key trait in biomechanical models of plant cells. Analysis of cell wall anisotropy in both barley and onion further highlighted the differences between dumbbell and kidney GC morphologies, agreeing with the consensus found by other studies (Shtein *et al*., 2017). Thus, barley GCs showed anisotropy with a predominantly longitudinal arrangement of cell wall microfibrils (Fig. S2a,b) and, since cellulose microfibrils are the major load‐bearing component of the plant cell wall, likely orientation of material stiffness. In onion GCs, anisotropy in the cell wall was also observed, supporting the previously suggested circumferential arrangement of cellulose microfibrils as found in other kidney‐shaped GCs (Fig. S2e,f).

### An FEM model of a grass stomate predicts that loss of synchronised GC inflation results in a decreased pore aperture per increment in turgor pressure

**Fig. 2:**
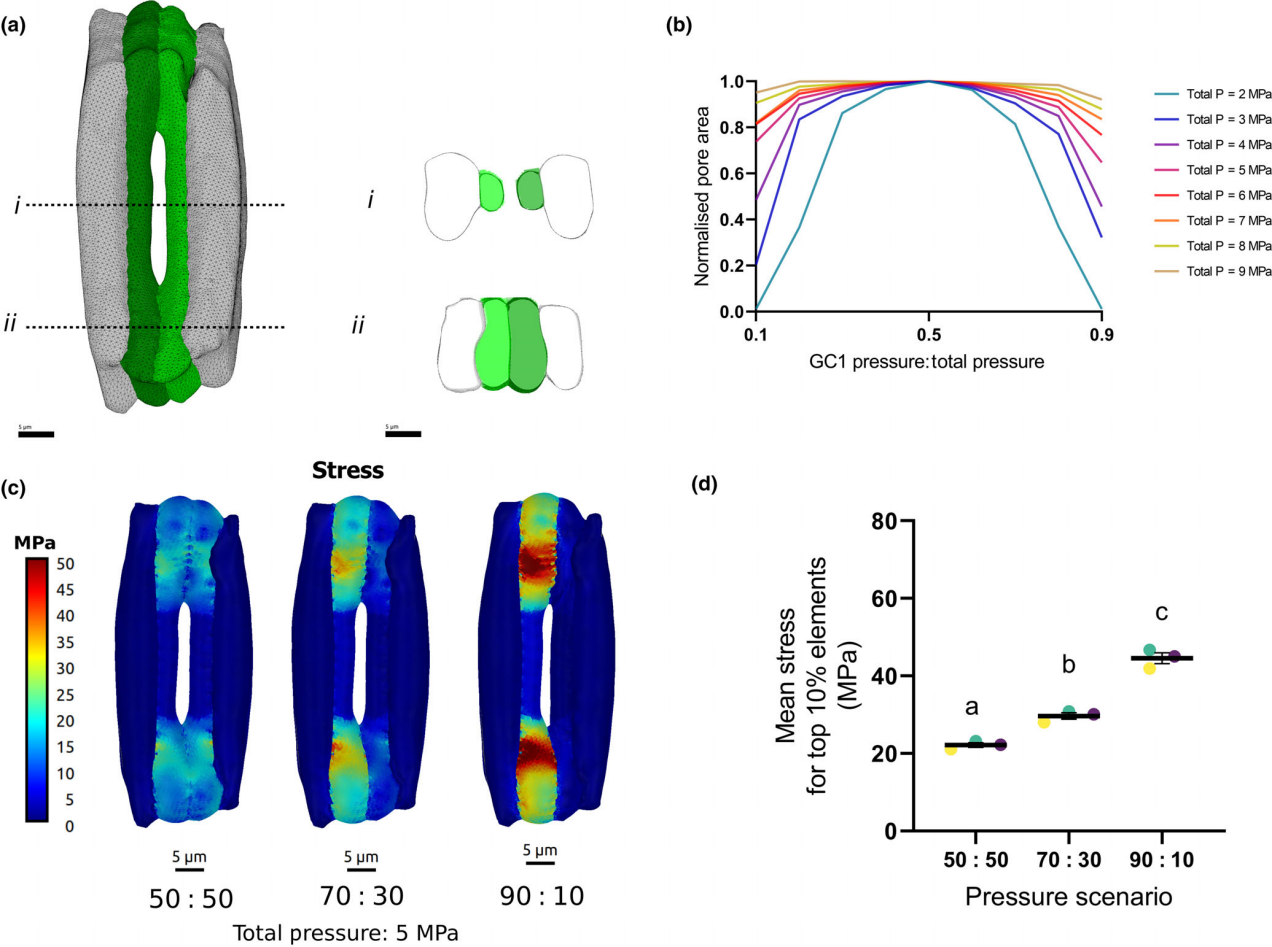
A Finite Element Method (FEM) model of grass stomata shows that synchronous guard cell (GC) pressure changes optimise pore opening. (a) 3D rendering of a barley (*Hordeum vulgare*) stomatal complex showing GC (green) and subsidiary cells (white/grey). Individual constituent cells of the stomatal complex were segmented from confocal image stacks to generate a mesh discretized into elements. Cross sections through the mesh in the (*i*) rod and (*ii*) bulbous end regions show the variation in cell wall thickness and shape. Bars, 5 μm. (b) Computational modelling of pore aperture at a range of guard cell pressures show that equal inflation of the two GCs results in optimal opening. As the modelled pressure in GC1 approaches 50% (0.5) of total pressure GC1 + GC2 (*x*‐axis), the normalised pore aperture (*y*‐axis) reaches a maximum. This is most apparent when total pressure in the system is low (teal, blue, purple lines, equating to 2, 3, 4 MPa, respectively). (c) Stress patterns for a barley stomatal complex. Left panel shows a stomate with a 50 : 50 (0.5 : 0.5) symmetric split of 5 MPa pressure. Centre panel shows a stomate with a 70 : 30 (0.7 : 0.3) asymmetric split of 5 MPa of total GC pressure between the two GCs. Right panel shows a stomate with a 90 : 10 (0.9 : 0.1) asymmetric spilt of 5 MPa pressure. When pressure is asymmetric, the GC with higher pressure has relatively high stress and its deformation is solely responsible for stomatal opening. The greater the pressure imbalance, the greater the stress. In a stomatal complex that is able to equalise pressure, the stress is equally shared among the GC pair, each GC has reduced stress and each GC contributes equally to pore area increase. The scale bar range was chosen to show maximum variation and allow comparison between models. Yellow and red colours show areas where the model indicates high stress occurs and darker blue areas indicate lower stress. Bars, 5 μm. (d) Quantification of the average stress for the top 10% of elements. Calculation of average stress supports the visualisation of stress patterns. Mean stress is higher in simulations of more unbalanced pressure ratios (one‐way ANOVA, *F*_(2,6)_ = 128.7, *P* ≤ 0.0001). GCs from the same biological replicate can be identified by colour. Differences between pressure scenarios were identified using *post hoc* Tukey's HSD with identical lettering indicating samples that cannot be distinguished from each other (*P* < 0.05). Error bars = SEM.

To investigate how the connections found in grass stomata influence stomatal mechanics, we ran simulations with our previously published 3D computational FEM model representative of grass stomatal function (Durney *et al*., 2023). Confocal light microscopy was used to collect image stacks of the four‐celled barley stomatal complex stained with propidium iodide. Barley stomata were segmented into individual GC and adjacent subsidiary cells, which enabled the creation of a 3D mesh of individual elements describing stomatal cell shape (Fig. 2a). Meshes of cell geometry, together with the analysis of cell wall anisotropy, cell wall thickness, and other literature derived parameters (Table S1) were used to parametrise the FEM model (Fig. S2).

**Fig. 3:**
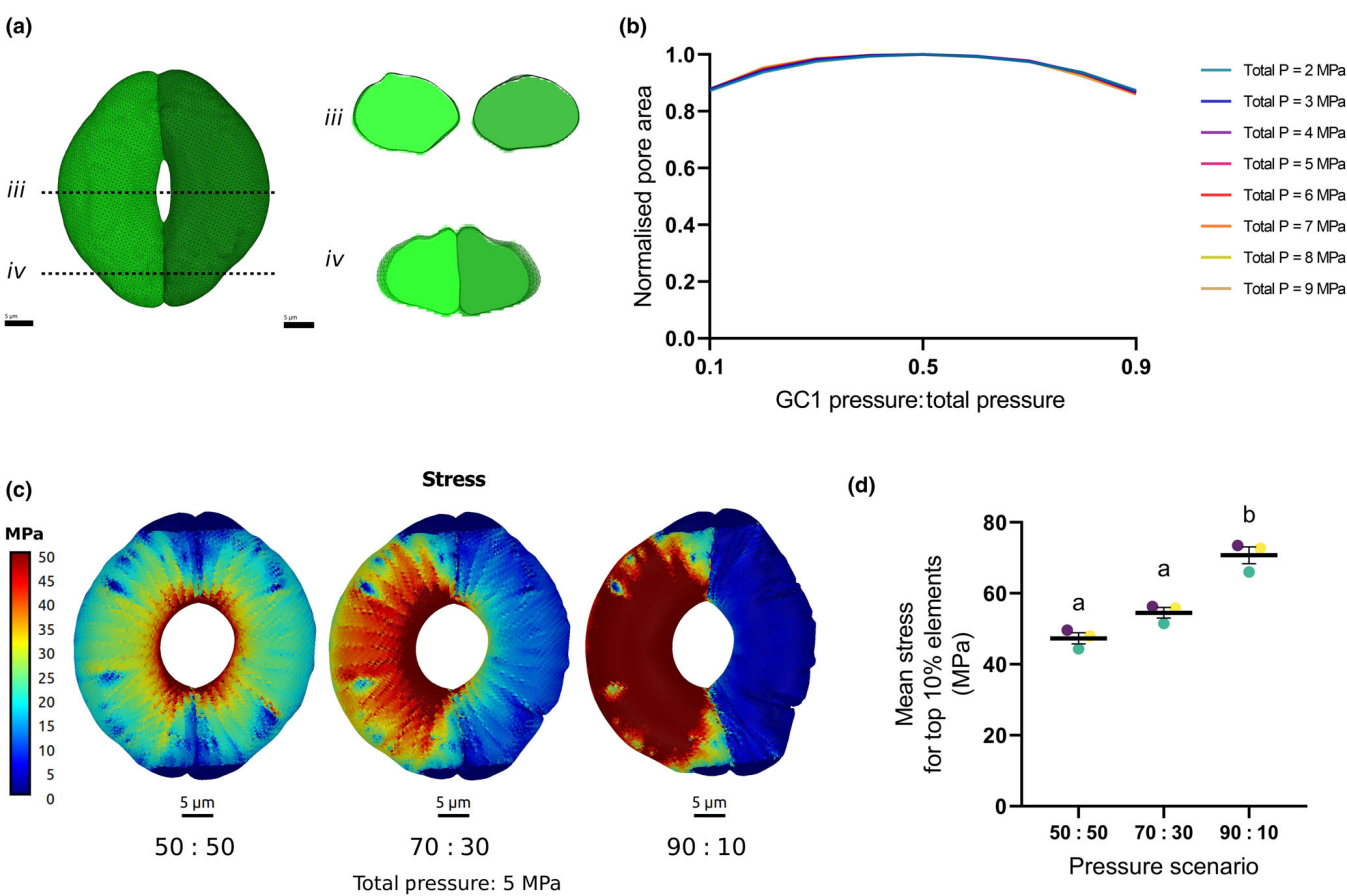
A Finite Element Method (FEM) model of onion stomatal function shows that asynchronous pressure changes have little impact on pore opening. (a) 3D rendering of an onion (*Allium cep*) stomatal complex showing the guard cell (GC) pair. Individual GCs (green) were segmented from confocal image stacks to generate a mesh discretized into elements. Cross sections across the centre of the mesh (*iii*) and towards the poles of the mesh (*iv*) are shown. Bars, 5 μm (b) Computational modelling of pore aperture at a range of kidney‐shaped guard cell pressures shows that pore area is minimally affected by unequal GC inflation. As the modelled pressure in GC1 approaches 50% (0.5) of total pressure GC1 + GC2 (*x*‐axis), the normalised pore aperture (*y*‐axis) reaches a maximum. Asymmetry in the pressure distribution between GC1 and GC2 leads to some decrease in pore aperture, but even under extreme pressure differences (0.2 or 0.8 ratios), the stomata are still capable of opening to over 90% of the maximum pore area. This effect is consistent across the different pressure ranges tested (1–9 MPa). (c) Stress patterns for the onion stomatal complex with or without pressure asymmetry. Left panel shows a 50 : 50 (0.5 : 0.5) split of the pressure between component GCs. Centre panel shows a GC pair with a 70 : 30 (0.7 : 0.3) split of 5 MPa of total pressure between the two component GCs. Right panel shows a stomate with a 90 : 10 (0.9 : 0.1) asymmetric spilt of 5 MPa pressure. When the pressure is unequal, the GC with higher pressure has increased stress; however, both cells are still able to contribute to pore area increase. In a stomatal complex that is able to equalise pressure (left hand image), the stress is equally shared among the component GCs, with each cell contributing equally to pore area. When pressure is asymmetric, the more highly pressurised GC has a greater contribution to stomatal opening. This becomes more disproportionate as pressure scenarios become more extreme. The scale bar range was chosen to show maximum variation and allow comparison between models. Yellow and red colours show areas where the model indicates high stress occurs and darker blue areas indicate lower stress. Bars, 5 μm. (d) Quantification of the mean stress for the top 10% of elements. Calculation of average stress supports the visualisation of stress patterns. Average stress is higher in simulations of more unbalanced pressure ratios (one‐way ANOVA, *F*_(2,6)_ = 41.74, *P* = 0.0003). GCs from the same biological replicate can be identified by colour. Differences between pressure scenarios were identified using *post hoc* Tukey's HSD with identical lettering indicating samples that cannot be distinguished from each other (*P* < 0.05). Error bars = SEM.

We hypothesised that the discontinuous wall between the adjoining GCs of grass stomata might act to equalise the internal turgor pressure within the GC pair, thus ensuring that they behave as one osmotic unit. To determine whether coordinated pressure changes benefit stomatal dynamics, we used the model to investigate a range of turgor pressures in individual guard cells (i.e. explore what would happen to stomatal aperture if there was or was not a mechanism for pressure equalisation). We kept the combined pressure of both GCs constant (the pressure of GC 1 plus GC 2) and varied the ratio of their individual pressures from 0.1 : 0.9 to 0.9 : 0.1. A ratio of 0.5 : 0.5 represented equal GC pressures, a scenario that would occur if symplastic GC connections serve to equalise turgor pressure. Each of the additional ratios explored was representative of GC isolation and their operation as individual pressure units. The total pressures examined ranged from 2 to 9 MPa. For each total GC pressure, the pore area resulting from the model was normalised against the maximum calculated pore area achieved for that pressure (Fig. 2b). Raw pore apertures are presented in Fig. S3(a). When the ratio of GC pressures was set to equal (0.5 : 0.5), the simulated pore area was maximal (indicated by a normalised pore area of 1.0). As the ratio of GC pressures was adjusted to be more unbalanced, a decrease in pore area was observed for the same total GC pressure. This effect was most pronounced at lower total guard cell pressure (e.g. 2–3 MPa) where there was a relatively large decrease in normalised pore area with asynchronous turgor pressure. For example, with a total pressure of 2 MPa (teal line in Fig. 2b), an asymmetric pressure accumulation where GC pressure is 0.3 of the total leads to the pore reaching only 85% of the aperture when pressure is equalised (0.5 : 0.5). At higher total pressure (7–9 MPa), the effect was less dramatic owing to one of the GCs becoming overinflated (beyond the physiological range) and overcompensating for the lack of pressure in the adjoining guard cell. Observations of the computed stress and strain patterns show a potential further mechanical benefit to the imposition of equal GC pressures. By equalising pressure within a GC pair, a greater pore area is achieved with lower stress (Fig. 2c) and strain (Fig. S4b) throughout the GCs. Quantification of average stress for the top 10% of elements showed that, for the mesh presented in Fig. 2(c), when GC pressure is equalised (0.5 : 0.5), the mean stress is 21.2 MPa. By contrast, when pressure is unequal with a ratio of 70 : 30 (0.7 : 0.3), the average stress increases to 26.97 MPa. More extreme simulations (90 : 10/0.9 : 0.1) increased the mean stress to 39.95 MPa. When this analysis was extended to include a range of meshes across three biological replicates, it was found that the balance of pressure had a significant impact upon the mean stress of the top 10% of elements (one‐way ANOVA, *F*_(2,51)_ = 128.7, *P* ≤ 0.0001; differences distinguished using *post hoc* Tukey's HSD) (Fig. 3d). Therefore, the presence of GC symplastic connections allows greater pore areas, and consequently (theoretically) increased leaf gas exchange rates, to be achieved at lower total GC pressure and with less mechanical stress on the system. Finally, analysis of actual GC geometry in confocal images of plant material suggests that, perhaps unsurprisingly, one GC is always larger in volume than the other within a pair (paired *t*‐test, *t*_(23)_ = 7.764, *P* ≤ 0.0001) (Fig. S5a). Similar patterns are observed when comparing additional geometrical parameters including surface area (Wilcoxon matched‐pairs signed‐rank test, *P* ≤ 0.0001) (Fig. S5c) and surface area to volume ratio (SA/V) (paired *t*‐test, *t*_(23)_ = 6.494, *P* ≤ 0.0001) (Fig. S5e). In the grass stomatal system, on average, the two GCs of each pair differed in volume by *c*. 9%. Differences within the pair for both surface area and SA/V were *c*. 5% and 4%, respectively.

### An FEM model predicts that whilst synchronised inflation of kidney‐shaped GCs is more efficient, this is not as dramatic as in dumbbell‐shaped GCs

To explore whether asynchronous inflation of GCs has different implications for stomatal dynamics with alternate geometries, we created a similar FEM model of stomatal function for onion, which displays kidney‐shaped GCs arranged in linear arrays comparable to grasses. Confocal light microscopy was again used to collect image stacks of onion stomata stained with PI. As for the barley model, images were segmented and meshes describing 3D GC geometries (Fig. 3a) were used to parameterise the model, alongside cell wall anisotropy data (Fig. S2) and mechanical properties from the literature (Table S1). As with previous mechanical models of kidney‐shaped stomata (Woolfenden *et al*., 2017; Yi *et al*., 2018), increasing turgor pressure in GCs results in an increased pore area (Fig. S3). The same parameter space of asynchronous GC pressures was simulated as performed for the barley model described previously (Figs 3b, S4c). In contrast to the barley model, although an optimum pore opening was observed when sister GC pressures were equal (equivalent to the single osmotic unit), the decline in stomatal performance at more unbalanced guard cell pressure ratios was less pronounced than in grass GCs (compare Fig. 3b vs Fig. 2b). For example, with a total pressure of 2 MPa (teal line in Fig. 3b), an asymmetric pressure accumulation where GC1 pressure is 0.3 of the total leads to the pore reaching over 97% of the aperture when pressure is equalised (0.5 : 0.5). In modelled onion stomata, a pore area > 80% of the maximum was still achieved even under the most extreme imbalanced GC pressure scenarios. When the stress (Fig. 3c), and strain (Fig. S4d) profiles of the kidney‐shaped GCs were analysed, the less pressurised GC still showed a region of high stress on the interior cell wall abutting the pore, a feature not seen in grass stomata. Comparison of the average stress for each pressure scenario showed a similar pattern to that observed in the barley simulations. For the mesh presented in Fig. 3(c), when pressure was equal across the GC pair, average stress was 49.9 MPa, whereas this was higher in more imbalanced pressure scenarios (0.7: 0.3 = 61.1 MPa; 0.9: 0.1 = 79.9 MPa). This pattern was found to be consistent for simulations across each of the onion meshes, with increased disparity in GC pressure resulting in a higher mean stress, although the 50 : 50 and 70 : 30 pressure scenarios could not be statistically distinguished from one another (one‐way ANOVA, *F*_(2,6)_ = 41.74, *P* = 0.0003; differences distinguished using *post hoc* Tukey's HSD) (Fig. 3d). As in barley stomata, when actual GC volumes within a pair were compared in the onion meshes (Fig. S5b), they were unequal (paired *t*‐test, *t*_(17)_ = 6.762, *P* ≤ 0.0001). Trends observed in surface area (paired *t*‐test, *t*_(17)_ = 5.488, *P* ≤ 0.0001) (Fig. S5d) and SA/V (paired *t* test, *t*_(17)_ = 5.163, *P* ≤ 0.0001; Fig. S5f) within the onion GC pair were similar to those shown in barley. However, the average difference in GC volume was *c*. 6% (Fig. 3d). Mean difference in GC surface area was *c*. 5%, and average difference in GC SA/V was *c*. 2.5%. Comparison of the mean percentage difference in SA/V within the GC pair was significantly greater in barley than in onion (unpaired *t*‐test, *t*_(40)_ = 2.549, *P* = 0.0147; Fig. S5g).

## Discussion

The presence of large gaps in the walls of adjoining GCs in grasses was initially observed over 50 yr ago, but these observations, let alone the functional importance of GC connections, seem to have been largely ignored. Initial work raised the hypothesis that stomata acting as a single osmotic unit would work more effectively (Kaufman *et al*., 1970; Srivastava & Singh, 1972; Voss *et al*., 2018), but analysis to support this idea has been lacking. In addition, the reasoning behind why connections are more frequently observed between dumbbell‐shaped rather than kidney‐shaped GCs has not been explored. The results presented here support the hypothesis that dumbbell‐shaped stomata open more effectively if they act as a single osmotic unit. The gaps observed in the shared ventral walls provide a mechanism whereby neighbouring GCs can rapidly equalise their individual turgor pressures. In the absence of such turgor equalisation, grass GCs run the risk of unequal turgor leading to a suboptimal mechanical response (cell shape change), leading to suboptimal pore opening, especially at low turgor pressures equating to the early phase of stomatal opening. By contrast, although our model indicates that kidney‐shaped GCs of species which lack such a turgor pressure equilibration mechanism, also achieve maximal pore area when the GCs have equal turgor pressure, unequal pressure in adjacent GCs results in a much smaller penalty in terms of suboptimal pore opening.

This difference in the importance of a pressure equalising system is due to the specialised geometry and opening mechanism of grass stomata, where GCs have stiffened central rod regions and bulbous dumbbell ends that push against each other to open the pore (Durney *et al*., 2023; Gkolemis *et al*., 2023). At the highest pressures simulated in our model, overinflation of one dumbbell end compensated for the lack of geometrical change in the other GC, but at lower and more physiologically relevant pressures, such mechanical compensation did not occur. This suggests that the connections between dumbbell‐shaped GCs are most useful at lower pressure ranges, equating to the phase when stomata are just beginning to respond to environmental triggers. The ability to effectively respond to a fluctuating environment is understood to have been a key evolutionary driver for the acquisition of the dumbbell‐shaped GCs, and in the success of grass species in populating the landmasses of the Earth (Hetherington & Woodward, 2003). Our data suggest that the advantage of dumbbell‐shaped GCs may only be fully realised if the GCs have gaps in their adjoining walls to allow pressure equilibration.

Observation of the stress and strain profiles of each GC revealed another potential benefit of equalising pressure within grass stomata. When kidney‐shaped GCs of a stomate are not equally pressurised in our simulations, for example a 70 : 30 split of turgor, the less pressurised GC still shows a region of high stress, similar in magnitude to that of the adjacent GC. However, in grass stomata simulations, this was not the case: the more pressurised guard cell had higher stress, whereas the other GC showed very little increase in stress levels over the closed state. For the kidney‐shaped stomata, in the most extreme asynchronous pressure scenarios (90 : 10), a more uneven distribution of stress between the GC pair was observed. In barley, when both GCs are under the same pressure, the stress is distributed equally between them, lowering the overall stress of the system for a given pore area. This would have multiple benefits for the grass system. It would minimise the risk of structural damage or plastic deformation of the cell wall as it undergoes repeated rounds of deformation and would require less energy input (i.e. to drive turgor) to achieve the maximal pore area.

Our biomechanical simulations indicate that stomata with kidney‐shaped GCs would also benefit from operating as a single osmotic unit, but not to the same degree as the dumbbell‐shaped GC system. Whilst there are examples of apparently symplastically connected kidney‐shaped GCs in the literature (Merced & Renzaglia, 2014; Cullen & Rudall, 2016; Voss *et al*., 2018), the data indicate that most species with kidney‐shaped stomatal morphology have GCs which are symplastically isolated once mature (Willmer & Sexton, 1979; Wille & Lucas, 1984; Zhao & Sack, 1999). This suggests some degree of plasticity in the system within which evolution might work. Whilst operating with balanced turgor pressure may be advantageous for rapidly moving grass stomata, being able to individually adjust GC pressure, and thus more precisely regulate pore aperture, may be more favourable in plants with slower stomatal responses. It is also plausible that the independent adjustments in GC pressure possible in kidney‐shaped eudicot GCs, and thus finer control over pore area, may provide an advantage in less humid environments over the connected kidney‐shaped guard cells found in some mosses and ferns (Merced & Renzaglia, 2014; Voss *et al*., 2018). Further exploration of the evolutionary distribution of symplastically connected kidney‐shaped GCs would inform on these hypotheses. Our analyses also suggest that, apart from under the most extreme asymmetrical pressure scenarios, isolated kidney‐shaped GCs both contribute considerably towards pore opening, and as such the requirements for balanced turgor may be less stringent.

Additionally, our data, albeit only from two species, suggest that the volume discrepancy between unconnected kidney‐shaped GCs within a pair (6%) is lower than observed in grasses (9%). Differences in GC surface area within a pair were more similar, being *c*. 5% for both species. Interestingly, the disparity in SA/V between individual GCs in a pair was greater in barley (5%) than onion (2.5%), which could be expected to have implications for solute exchange, and thus, the pressure‐driven shape changes needed for stomatal movement. Our simulations suggest that GCs operating as a single pressure unit (as enabled by the presence of gaps in the shared ventral wall in grass species) would negate any potential negative impacts of uneven cell size, and consequently unequal turgor, therefore allowing greater variation of GC size during stomatal differentiation. At present, there are essentially no data on how, mechanistically, the gaps in cell walls that provide connections between grass GCs are formed. Even basic knowledge on whether it occurs during the initial process of cytokinesis (as seems to occur in ferns) or is a postdivision process is unclear, and it is unknown how they may change with stomatal complex age. Given this lack of fundamental understanding, it is extremely difficult at present to experimentally test the hypotheses raised here. Our study emphasises the role that computational modelling can play in approaching such experimentally recalcitrant problems, providing plausible solutions and encouraging future lines of research. Since our results indicate that gain or loss of GC connections is unlikely to be lethal, a genetic approach seems feasible, but would require a fine quantitative phenotyping screen.

In conclusion, combined with their specific geometry, cell wall mechanical properties and a reciprocal pressure exchange system (Franks & Farquhar, 2007; Nunes *et al*., 2020), we propose that common turgor pressure within the GC pair, facilitated by gaps in the connected cell wall, is an evolutionary important adaptation required in grass GCs for the full acquisition of a superior stomatal performance over the kidney‐shaped GCs found in most eudicots and other plant clades.

## Competing interests

None declared.

## Author contributions

MJW, SM, RJM and AJF were involved in conceptualisation. MJW, SM, CHD, MT, JA and RSS were involved in investigation. MJW, CHD and MT were involved in writing – original draft. All authors were involved in writing – review and editing. AJF, JEG, RJM and RSS were involved in supervision. AJF, JEG, RJM and RSS were involved in funding acquisition.

## Disclaimer

The New Phytologist Foundation remains neutral with regard to jurisdictional claims in maps and in any institutional affiliations.

## Supporting information


**Fig. S1** Observation of guard cell connections using confocal microscopy.
**Fig. S2** Cell wall anisotropy and boundary conditions for barley and onion models.
**Fig. S3** Validation of onion stomata Finite Element Method model.
**Fig. S4** Raw pore areas and strain maps for barley and onion simulations.
**Fig. S5** Comparisons of guard cell (GC) geometry within GC pairs in barley and onion.
**Table S1** Parameters used in the Finite Element Method models of barley and onion stomata.

**Video S1** Observation of guard cell connections in barley using confocal microscopy.

**Video S2** Observation of absence of guard cell connections in onion using confocal microscopy.
Please note: Wiley is not responsible for the content or functionality of any Supporting Information supplied by the authors. Any queries (other than missing material) should be directed to the *New Phytologist* Central Office.

## Data Availability

Microscopy data reported in this paper are publicly available at: https://doi.org/10.5281/zenodo.14793472. All scripts used to run simulations and process data are publicly available at https://github.com/mtomtom/symplastic‐guard‐cell‐connections.
