# Supplementary material for: Symplastic guard cell connections buffer pressure fluctuations to promote stomatal function in grasses
Source: New Phytol. 2025 Feb 15;246(1):192–203. doi: 10.1111/nph.70009 (PMC11883054; doi:10.1111/nph.70009)
Supplement: Supplementary file 1 — Fig. S1 Observation of guard cell connections using confocal microscopy. Fig. S2 Cell wall anisotropy and boundary conditions for barley and onion models. Fig. S3 Validation of onion stomata Finite Element Method model. Fig. S4 Raw pore areas and strain maps for barley and onion simulations. Fig. S5 Comparisons of guard cell (GC) geometry within GC pairs in barley and onion. Table S1 Parameters used in the Finite Element Method models of barley and onion stomata. [file NPH-246-192-s001.pdf]

New Phytologist Supporting Information

Article title: Symplastic guard cell connections buffer pressure fluctuations to promote stomatal function in grasses

Authors: Matthew J. Wilson, Shauni McGregor, Clinton H. Durney, Melissa Tomkins, Jodie Armand, Richard S. Smith, Julie E. Gray, Richard J. Morris, Andrew J. Fleming

Article acceptance date: 20 January 2025

Table S1.

Parameters used in the FEM models of barley and onion stomata.

Parameter	Value	Source
Barley - Young's modulus		
E1-E3	40 MPa	(Durney et al. 2023)
E2	75 MPa	(Durney et al. 2023)
Barley - General parameters		
Poisson's Ratio, v	0.3	(Strauss et al. 2022)
GC Pressure	0.2-5 MPa	(Franks and Farquhar 2007)
SC Pressure	0.2	(Franks and Farquhar 2007)
Wall thickness, Rod region/BE	3.0	(Durney et al. 2023)
Onion - Young's modulus		
E1-E3	40 MPa	Inferred, this work
E2	400 MPa	Inferred, this work
Onion - General parameters		
Poisson's Ratio, v	0.3	(Strauss et al. 2022)
GC Pressure	0.2-5 MPa	(Franks and Farquhar 2007)
Wall thickness, Mediolateral/polar	1.0	(Carter et al. 2017)

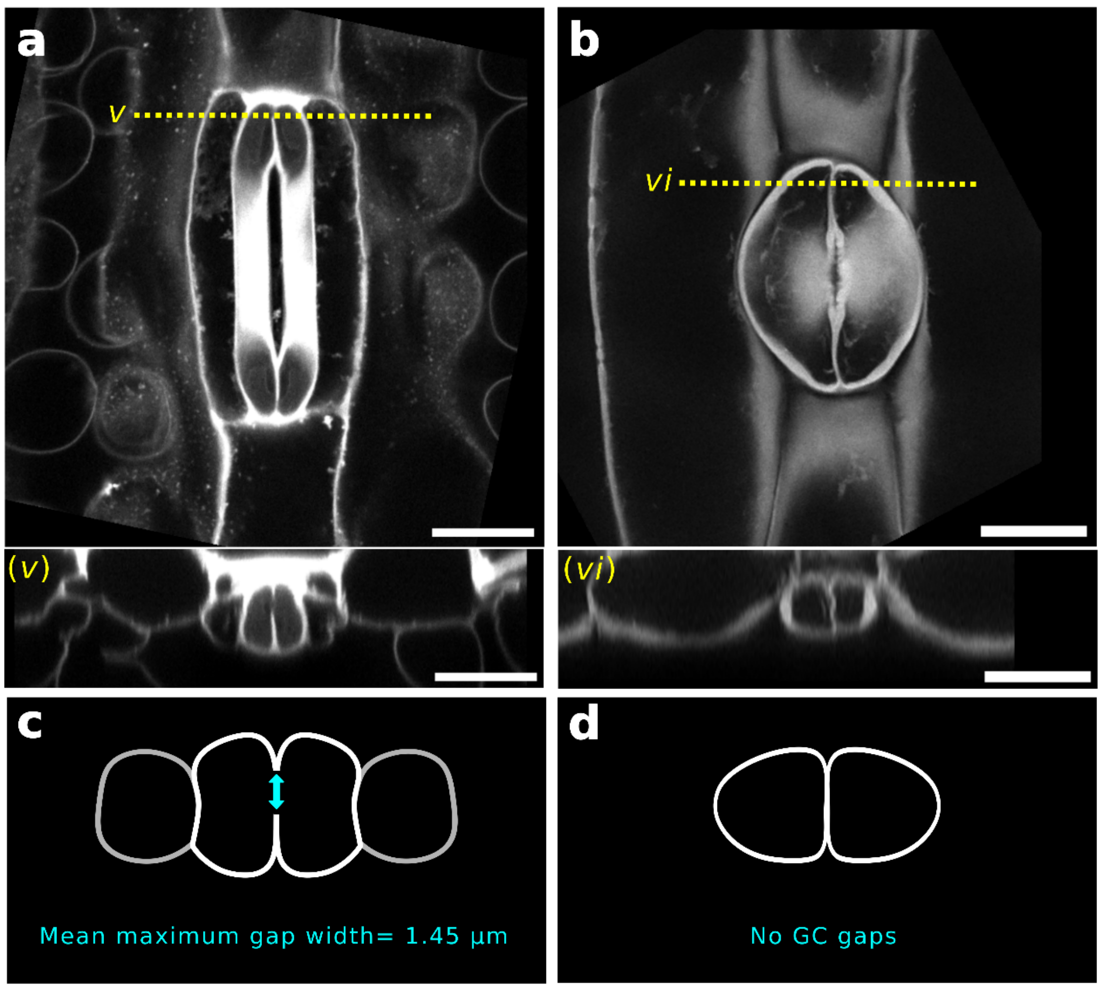

Figure S1. Observation of GC connections using confocal microscopy

(**a**) Analysis of confocal images used for mesh creation showed visible gaps in the bulbous ends of barley GCs. Dotted line shows the plane of transverse section. The incomplete shared cell wall is clearly visible. (**b**) Onion GCs are isolated from one another, with no visible large connections between the GC pair. Dotted line shows the plane of transverse section. (**c**) Cartoon summarising maximum GC gap width measurements in barley. The slice containing the maximum extent of the gap between guard cells (identified by a lack of signal between the two GCs) was identified after the image stack was resliced to show the XZ plane. Gap width in this slice was measured as highlighted by the cyan arrow. (**d**) Cartoon summarising that no GC connections could be observed in transverse section in onion stomata. Scale bars = 20 µm.

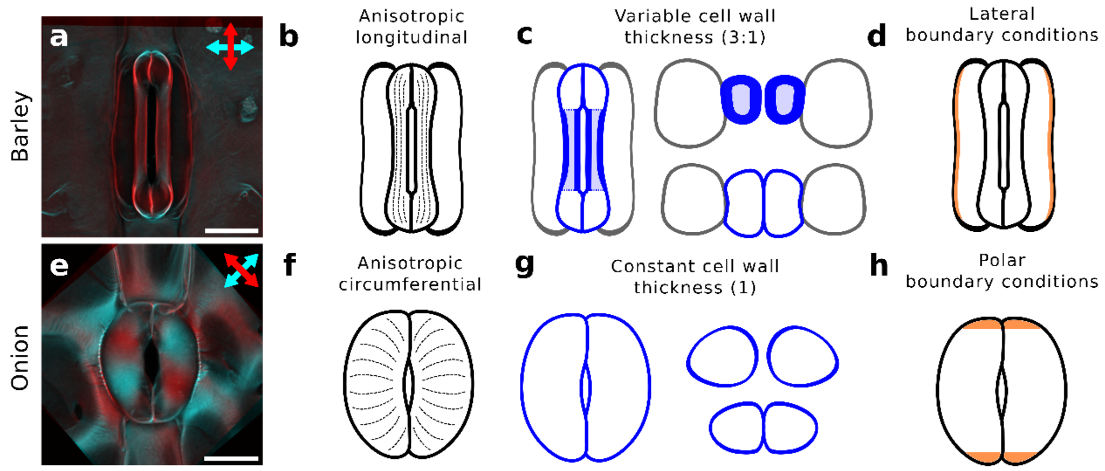

Figure S2. Cell wall anisotropy and boundary conditions for barley and onion models. (**a**) Analysis of cellulose orientation in barley stomata reveals anisotropy in the guard cell wall. Cellulose microfibrils (CMFs) are inferred to be primarily oriented longitudinal to the long axis of the GCs (red signal). CMFs oriented orthogonally to the GC long axis (cyan signal) are restricted to the poles of the complex at the bulbous ends. Scale bars = 20 µm. (**b**) Accordingly, cell wall anisotropy (dashed lines) is implemented in the barley model in a longitudinal fashion. (**c**) To take into account the variable wall thickness observed in grass GCs, cell wall thickness in the rod region was set at 3x that in the bulbous ends. (**d**) As barley stomata are arranged in files and complex width does not change with stomatal movement, lateral boundary conditions are applied to the edges (orange) of SC meshes in the barley model (**e)** Analysis of cellulose orientation in onion stomata reveals that CMFs are oriented in a circumferential arrangement (red and cyan signal). (**f**) In the onion model, cell wall anisotropy is implemented circumferentially (dashed lines). (**g**) Onion meshes are assumed to have constant wall thickness. (**h**) As polar fixation of kidney-shaped GCs is thought to be important for stomatal biomechanics (Carter et al. 2017), boundary conditions are enforced at the poles of GC meshes (orange) in the onion model.

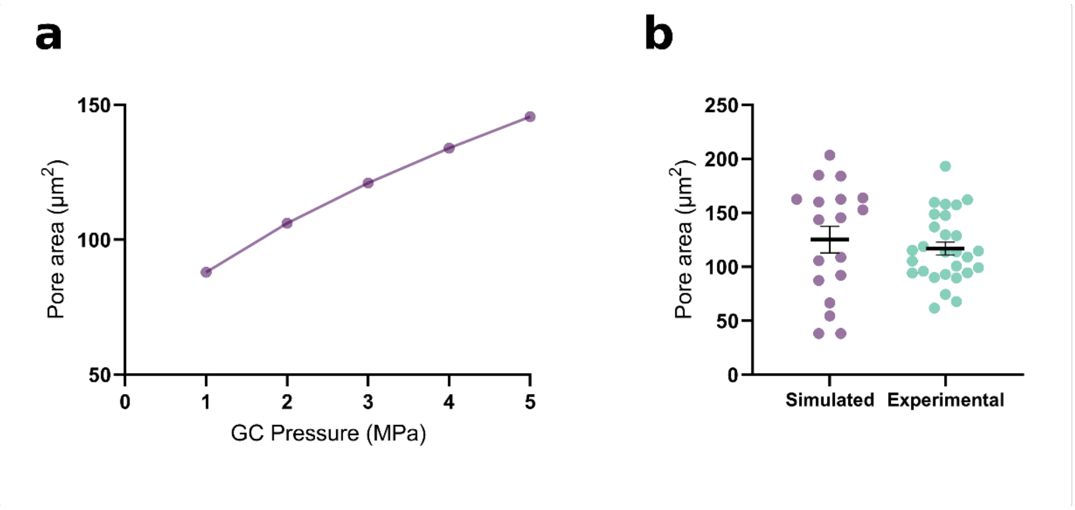

Figure S3. Validation of onion stomata FEM model.

(**a**) Pore aperture/ GC pressure response curve for an onion FEM model indicates that this successfully predicts kidney-shaped GC movements. (**b**). Comparison of experimental and simulated open pore area. The onion FEM model is able to simulate measured pore areas (Unpaired *t* test, $t_{(44)}$= 0.6682, *P*= 0.5075). Error bars = +/- 1 SEM.

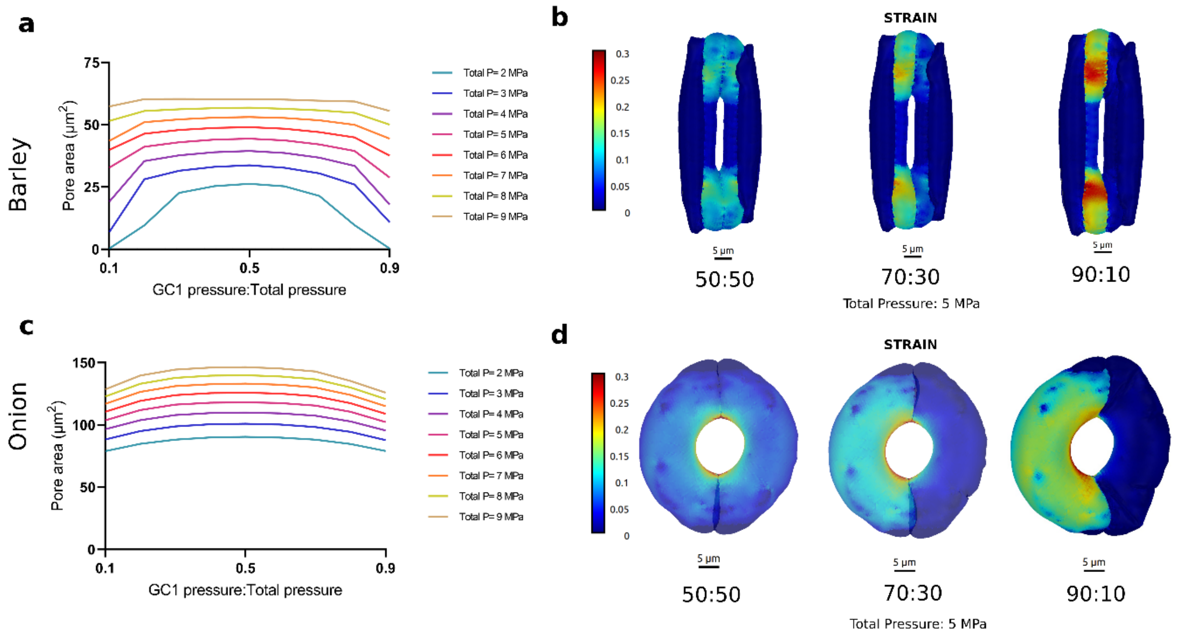

Figure S4. Raw pore areas and strain maps for barley and onion simulations.

(**a**) Computational modelling of barley pore aperture at a range of GC pressures shows that equal inflation of the two GCs results in optimal opening. As the modelled pressure in GC1 approaches 50% (0.5) of total pressure GC1 + GC2, pore area reaches a maximum. This is more pronounced at lower total pressures. (**b**) Strain pattern for a barley stomatal complex. When pressure is asymmetric, the guard cell with higher pressure has relatively high strain and its deformation is solely responsible for stomatal opening. In a stomatal complex that can equalise pressure, strain is equally shared among the guard cell pair, each guard cell has reduced strain, and each guard cell contributes equally to pore area increase. Scale bar range chosen to show maximum variation and allow comparison between species. Yellow and red colours show areas where the model indicates high strain occurs and darker blue areas indicate lower strain. (**c**) Computational modelling of onion pore aperture at a range of GC pressures shows that pore area is minimally affected by unequal GC inflation. As the modelled pressure in GC1 approaches 50% (0.5) of total pressure GC1 + GC2, the pore aperture reaches a maximum, but unbalanced pressure scenarios have little impact upon pore aperture. (**d**) Strain patterns for an onion stomatal complex. When the pressure is unequal, the guard cell with higher pressure has increased strain, however, both cells are still able to contribute to pore area increase. In a stomatal complex that can equalise pressure, the strain is equally shared among the component GCs, with each cell contributing equally to pore area. Yellow and red colours show areas where the model indicates high strain occurs and darker blue areas indicate lower strain.

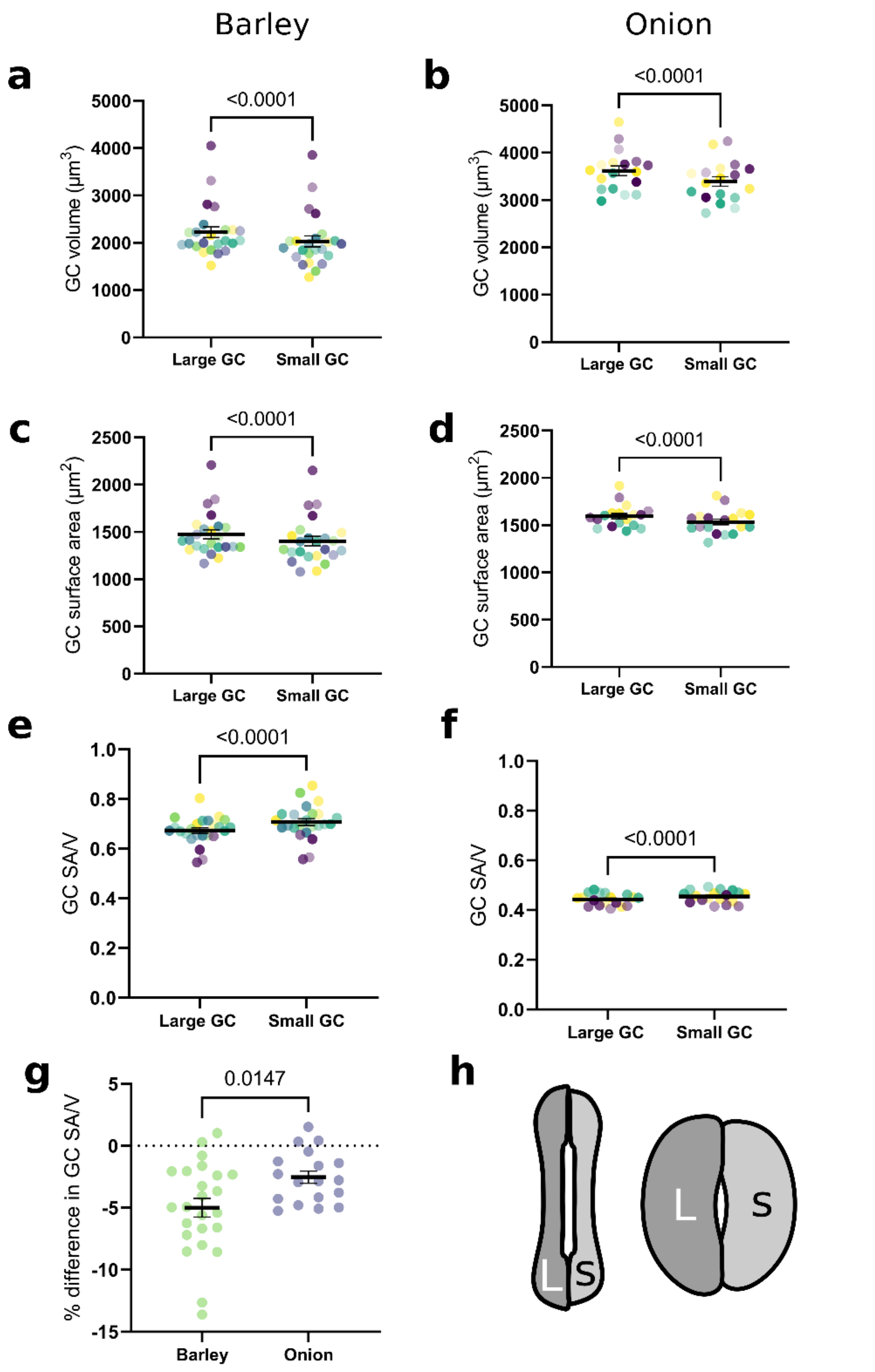

Figure S5. Comparisons of GC geometry within GC pairs in barley and onion

(**a**) Comparison between barley guard cell volumes measured from confocal stacked images of GC pairs. In each GC pair, one of the GCs has a greater volume than the other (paired *t* test, $t_{(23)}$= 7.764, *P*= <0.0001, n= 24 GC pairs). Guard cells within the same pair can be identified by colour. Error bars = +/- 1 SEM. (**b**) Comparison of onion guard cell volumes measured from confocal images (Paired *t* test, $t_{(17)}$= 6.762, *P*= <0.0001, n= 18 GC pairs). Guard cells within the same pair can be identified by colour. Error bars = +/- 1 SEM. (**c**) Mean barley GC surface area as quantified from confocal images. In each GC pair, one of the GCs

has a greater surface area than the other (Wilcoxon matched-pairs signed-rank test, *P*= <0.0001, n= 24 GC pairs). Guard cells within the same pair can be identified by colour. Error bars = +/- 1 SEM. (**d)** Onion GC surface area was found to be greater in one cell of the GC pair (Paired *t* test, $t_{(17)}$= 5.488, *P*= <0.0001, n= 18 GC pairs). Guard cells within the same pair can be identified by colour. Error bars = +/- 1 SEM. (**e**) Comparison of SA/V between barley GCs. In each GC pair, the smaller of the two GCs has a higher SA/V ratio (paired *t* test, $t_{(23)}$= 6.494, *P*= <0.0001, n= 24 GC pairs) Guard cells within the same pair can be identified by colour. Error bars = +/- 1 SEM. (**f**) Comparison of SA/V between onion GCs. In each GC pair, the smaller of the two GCs has a higher SA/V ratio (paired *t* test, $t_{(17)}$= 5.163, *P*= <0.0001, n= 18 GC pairs). Guard cells within the same pair can be identified by colour. Error bars = +/- 1 SEM (**g**) Comparison of the % difference in SA/V within the GC pair showed that this difference between the cells was larger in barley than onion (unpaired *t* test, $t_{(40)}$= 2.549, *P*= 0.0147). Error bars = +/- 1 SEM. (**h**) For both barley and onion, in each guard cell pair one GC is geometrically larger than the other (by default) small GC, as demonstrated (largest cell identified by the letter L, smallest by the letter s – not to scale).

Video S1. Observation of GC connections in barley using confocal microscopy

Video scrolling along the length of a barley stomatal complex in the XZ plane. PI signal shows the presence of cell wall material. Breaks in the shared ventral wall between the GC bulbous ends can be clearly identified. Scale bar = 20 µm.

Video S2. Observation of absence of GC connections in onion using confocal microscopy.

Video scrolling along the length of an onion stomatal complex in the XZ plane. PI signal shows the presence of cell wall material. No symplastic connections between the GC pair are visible. Scale bar = 20 µm.

Supplemental References

Carter R, Woolfenden H, Baillie A, Amsbury S, Carroll S, Healicon E, Sovatzoglou S, Braybrook S, Gray JE, Hobbs J, Morris RJ, Fleming AJ (2017) Stomatal Opening Involves Polar, Not Radial, Stiffening Of Guard Cells. Curr Biol 27:2974-2983 e2972. doi:10.1016/j.cub.2017.08.006

Durney CH, Wilson MJ, McGregor S, Armand J, Smith RS, Gray JE, Morris RJ, Fleming AJ (2023) Grasses exploit geometry to achieve improved guard cell dynamics. Curr Biol 33:2814-2822 e2814. doi:10.1016/j.cub.2023.05.051

Franks PJ, Farquhar GD (2007) The mechanical diversity of stomata and its significance in gas-exchange control. Plant Physiol 143 :78-87. doi:10.1104/pp.106.089367

Strauss S, Runions A, Lane B, Eschweiler D, Bajpai N, Trozzi N, Routier-Kierzkowska A-L, Yoshida S, Rodrigues da Silveira S, Vijayan A, Tofanelli R, Majda M, Echevin E, Le Gloanec C, Bertrand-Rakusova H, Adibi M, Schneitz K, Bassel GW, Kierzkowski D, Stegmaier J, Tsiantis M, Smith RS (2022) Using positional information to provide context for biological image analysis with MorphoGraphX 2.0. eLife 11:e72601. doi:10.7554/eLife.72601
